# Effects of paraprobiotics on bile acid metabolism and liver health in largemouth bass (*Micropterus salmoides*) fed a cottonseed protein concentrate-based diet

**DOI:** 10.1016/j.aninu.2023.02.011

**Published:** 2023-03-07

**Authors:** Xiaoze Xie, Xiaofang Liang, Hao Wang, Qiang Zhu, Junjun Wang, Ying Chang, Eric Leclercq, Min Xue, Jie Wang

**Affiliations:** a National Aquafeed Safety Assessment Center, Institute of Feed Research, Chinese Academy of Agricultural Sciences, Beijing 100081, China; b Xinjiang Jinlan Plant Protein Co., Ltd, Shihezi 833200, China; c State Key Laboratory of Animal Nutrition, College of Animal Science and Technology, China Agricultural University, Beijing 100193, China; d Lallemand SAS, Blagnac 31702, France

**Keywords:** Cottonseed protein concentrate, Multi-yeast strain fractions, *Micropterus salmoides*, Liver health, Bile acid metabolism, Gut microbiota

## Abstract

Cottonseed protein concentrate is a sustainable fishmeal alternative in aquafeed. A 10-week experiment was conducted to investigate the effects of a cottonseed protein concentrate-based diet with and without multi-strain yeast fractions (MsYF) on growth, bile acid metabolism, and health in largemouth bass. Four hundred fish (54.0 ± 0.0 g) were casually distributed into 16 tanks (4 replicates/diet). Fish were fed with 4 iso-nitrogen and iso-energetic diets 3 times daily, including a fishmeal diet (FM), a soy protein concentrate-based diet (SPC; replacing 81% fishmeal protein), a cottonseed protein concentrate-based diet (CPC; replacing 81% fishmeal protein), and a CPC diet supplemented with 800 mg/kg MsYF (CPCY). Results showed that the survival of SPC was the lowest, i.e., 48%, with no apparent diet effect among other treatments; we omitted the SPC in additional analyses. Fish fed cottonseed protein concentrate-based diets showed lower growth than FM (*P* < 0.05). Fish fed CPC showed the highest nuclear dense hepatic phenotypes ratio (50%), followed by CPCY (33%) and FM (17%). Further, dietary CPC increased hepatic total cholesterol and triglyceride levels with concurrently increased cholesterol synthesis but decreased triglyceride synthesis-associated transcription levels (*P* < 0.05). Furthermore, dietary CPC increased bile acid synthesis but decreased bile acid transport-associated transcription levels (*P* < 0.05), and then induced an increment of plasma cholic acid and hepatic chenodeoxycholic acid content and the decrement of genus *Romboustia* (*P* < 0.05). Regarding the effect of MsYF, fish fed CPCY reduced hepatic lipid accumulation and total plasma bile acid content (*P* < 0.05) compared to CPC, suggesting an improvement in liver health. Also, dietary MsYF could reverse the microbiota community structure showing a similar gut microbial composition to FM. In conclusion, 81% of fishmeal protein replaced by cottonseed protein concentrate suppressed growth and liver health, while dietary MsYF might mitigate the negative impact of a high cottonseed protein concentrate level diet on liver functions via gut microbiota regulation.

## 1 Introduction

Largemouth bass (*Micropterus salmoides*) originated from North America and has been introduced as a commercial fish species worldwide owing to its rapidly growing and valuable market (Tidwell et al., 2005). In China, largemouth bass is widely cultured, and its output increased to 0.62 million tonnes in 2020 (Xi et al., 2022). As a carnivorous fish, the appropriate dietary protein requirement of largemouth bass has been recommended to be 48% to 52%, with fishmeal as the primary protein source (Cai et al., 2020). Fishmeal is aquafeed's top important protein ingredient (Gatlin et al., 2007), but its price has been pushed up due to finite wild-captured forage fish resources, which has stressed the expanding global aquaculture industry (FAO, 2018). Hence, developing a novel non-food fishmeal alternative is crucial to meet the demands of the growing aquaculture industry (Wang et al., 2022a).

Plant protein sources as fishmeal alternatives are competitively priced and have stable production. As a common food ingredient, soy protein concentrate has been well utilized in aquafeed, but it has several obvious limitations, like the presence of anti-nutritional factors (ANFs) and competing as a food resource with humans (Carral et al., 2021; Chen et al., 2019). Cottonseed, as a crucial non-food ingredient, has been estimated to be 43.8 million tonnes globally in 2021/2022 (Statista, 2023), which has the potential to produce 8.8 million tonnes of protein (Kumar et al., 2021). However, cottonseed has several ANFs, especially gossypol, which adversely affects fish health resulting in its limited use in aquafeed. Nowadays, cottonseed can be processed into cottonseed protein concentrate characterized with high protein content and lower gossypol via advanced processing technology (Xie et al., 2022). Previous studies have showed that cottonseed protein concentrate replacing less than 50% of fishmeal protein did not affect the growth of *Trachinotus ovatus* (Shen et al., 2020), *Oncorhynchus mykiss* (Zhao et al., 2021), *Sillago sihama* Forsskál (Liu et al., 2020), *Sciaenops ocellatus* (Wang et al., 2020), *Epinephelus fuscoguttatus*♀ × *Epinephelus lanceolatus*♂ (Yin et al., 2018), *Centropristis striata* (Anderson et al., 2016), and *M. salmoides* (Liu et al., 2021; Wang et al., 2021b; Xie et al., 2022; Xu et al., 2022). Higher than 50% substitution level by cottonseed protein concentrate could adversely affect the growth and even induce intestinal inflammation in *S. sihama* Forsskál (Liu et al., 2020) and hybrid grouper (Yin et al., 2018). Also, Liu et al. (2021) reported that the 75% cottonseed protein concentrate substitution level suppressed growth and damaged liver health, accompanied by lipid accumulation in largemouth bass (Liu et al., 2021). Nevertheless, another study on largemouth bass suggested that the 60% to 80% cottonseed protein concentrate substitution level did not negatively influence growth (Wang et al., 2021b). Thus, it is still controversial as to which is the suitable fishmeal substitution level for largemouth bass.

In our recent study, the 40% fishmeal protein substitution level by cottonseed protein concentrate promoted the largemouth bass’ growth (Xie et al., 2022). Multi-strain yeast fraction (MsYF) supplementation in the cottonseed protein concentrate-containing diet could further enhance growth and improve intestinal health (Xie et al., 2022). Of note, the MsYF combines 3 selected yeast fractions from 2 *Saccharomyces* *cerevisiae* strains and a *Cyberlindnera* *jadinii* strain and contains 45% total polysaccharides, which could activate distinct immune pathways, including pro- and anti-inflammatory cytokines (Rawling et al., 2019, 2021; Rawling et al., 2019). Dietary MsYF promoted the immune response and intestinal surface area, thus improving nutrient absorption in fish (Rawling et al., 2019, 2021; Rawling et al., 2019). Additionally, dietary yeast-based paraprobiotics have been found to regulate intestinal microbiome composition, increase fecal bile acid excretion, decrease blood cholesterol content, and migrate lipid accumulation (Feng et al., 2022; So et al., 2021).

Collectively, this study aims to compare the effect of soy protein concentrate or cottonseed protein concentrate as fishmeal substitutions (81% fishmeal protein) on the growth, liver health, and intestinal microbiome of largemouth bass, and whether MsYF supplementation could benefit the growth and health of largemouth bass fed a cottonseed protein concentrate-based diet.

## 2 Material and methods

### 2.1 Animal ethics statement

In our experiment, all fish complied with the Laboratory Animal Welfare Guidelines in China (Decree No. 2 of Ministry of Science and Technology, issued in 2021).

### 2.2 Diets

In the study of Xie et al. (2022), the 40% fishmeal substitution by cottonseed protein concentrate improved the growth of largemouth bass. Hence, we further increased the cottonseed protein concentrate substitution level to 81% fishmeal protein in this study. Table 1 shows the feed materials and nutrition composition of each diet. Four iso-nitrogenous and iso-energetic practical diets were formulated: 1) a fishmeal diet (53% fishmeal; FM); 2) a soy protein concentrate diet (10% fishmeal and 43.2% soy protein concentrate; SPC); 3) a cottonseed protein concentrate diet (10% fishmeal and 42% cottonseed protein concentrate; CPC) and 4) the CPC diet supplemented with 800 mg/kg MsYF (CPCY). The MsYF combines selected yeast fractions from 2 *S. cerevisiae* strains and a *C. jadinii* strain, containing 45% total polysaccharides. The biochemical, biophysical, and specific immune properties of this proprietary product (Lallemand SAS; Blagnac, France) are described elsewhere (Rawling et al., 2019; Schiavone et al., 2017). Three amino acids (lysine, methionine, and threonine) were added to each diet to fulfill the amino acid requirements for largemouth bass, whose amino acid composition is shown in Table S1. The diets were processed into floating pellets (3-mm diameter) by these sections: feeding process (90 °C, 5 s), compression process (90 to 108 °C, 5 s), and metering process (94 to 112 °C, 4 s) by using a Twin-screwed extruder. Each diet was dried, cooled after being vacuum-coated at room temperature with fish oil, and stored at 4 °C.

**Table 1:** Formulation and composition of the experimental diets.^1^

Item	FM	SPC	CPC		CPCY
Ingredients, g/kg as-is basis					
Fish meal^2^	530	100	100		100
Cottonseed protein concentrate^3^	–	–	420		420
Soy protein concentrate^4^	–	432	–		–
Soybean meal^4^	100	100	100		100
Wheat gluten^4^	40	40	40		40
Spray-dried blood cell powder^5^	50	50	50		50
Wheat flour^6^	50	50	50		50
Tapioca starch^6^	50	50	50		50
Alpha-cellulose^7^	80	0	6.1		5.3
Kelp powder^8^	15	15	15		15
Ca(H_2_PO_4_)_2_^9^	–	24.7	24.7		24.7
Lecithin oil^4^	20	20	20		20
Fish oil^10^	20.2	40.2	40.2		40.2
Soybean oil^4^	30	52	45		45
Vitamin and mineral premix^11^	13.8	13.8	13.8		13.8
L-Lys·HCl^12^	–	6.1	15.7		15.7
DL-Met^12^	–	4.2	3.6		3.6
L-Thr^12^	–	1	4.9		4.9
Multi-strain yeast fractions (MsYF)^13^	–	–	–		0.8
Y_2_O_3_^14^	1	1	1		1
Total	1000	1000	1000		1000
Proximate analysis, % dry matter basis					
Dry matter	96.62	96.75		95.71	97.76
Crude protein	51.38	52.13		51.72	51.28
Crude lipid	11.37	12.04		12.20	12.20
Crude ash	12.21	9.19		9.42	9.39
Crude fiber + Nitrogen free extract	25.04	26.64		26.66	27.14
Gross energy, MJ/kg	20.84	21.29		21.14	21.89

1 FM = fish meal diet; SPC = soy protein concentrate diet; CPC = cottonseed protein concentrate diet; CPCY = CPC diet + 800 mg/kg multi-strain yeast fractions (MsYF).

2 Supplied by Tecnológica de Alimentos S.A., Ltd. (Peru): 67% crude protein.

3 Supplied by Xinjiang Jinlan Vegetable Protein Co. Ltd. (China): 64% crude protein, 179 mg/kg free gossypol which was less than the toleration limitation of free gossypol content (≤400 mg/kg) in Hygienical Standard for Feeds (GB 13078-2017).

4 Supplied by Bohai Oil Co., Ltd. (China): 64% crude protein.

5 Supplied by Beijing Hongshun Source Biotech Co., Ltd. (China).

6 Supplied by Haid Group Co., Ltd. (China).

7 Supplied by Huzhou City Linghu Xinwang Chemical Co., Ltd. (China).

8 Supplied by Qingdao Hisea Imp. & Exp. Co., Ltd. (China).

9 Supplied by Yunnan Phosphate Group Co., Ltd. (China).

10 Supplied by Jinhai Grain & Oil Industry Co., Ltd. (China): Fish oil containing antioxidant - tertiary butylhydroquinone (TBHQ; 200 mg/kg diet).

11 Vitamin and mineral premix (mg/kg diet): vitamin A 20; vitamin B_1_ 10; vitamin B_2_ 15; vitamin B_6_ 15; vitamin B_12_ (1%) 8; niacinamide 100; vitamin C phosphate (35%) 1000; calcium pantothenate 40; biotin (2%) 2; folic acid 10; vitamin E (50%) 400; vitamin K_3_ 20; vitamin D_3_ 10; inositol 200; corn protein powder 150. FeSO_4_·H_2_O 300; ZnSO_4_·H_2_O 200; MnSO_4_·H_2_O 100; KI (10%) 80; Na_2_SeO_3_ (10% Se) 10; CoCl_2_·6H_2_O (10% Co) 5; NaCl 100; MgSO_4_·5H_2_O 2000; zeolite 5005; choline chloride 4000.

12 Supplied by Beijing Enhalor International Tech Co., Ltd. (China).

13 Supplied by Lallemand SAS. (France).

14 Supplied by Sinopharm Chemical Reagent Co., Ltd. (China).

### 2.3 Fish, system and set-up

A 10-week experiment (70 days) was performed in the Nankou research area (Institute of Feed Research, Beijing, China). Largemouth bass were acquired from a fish farm (Tianjin Yuqing Aquatic Technology Company, Tianjin, China), and adapted in a recirculated tank system for 14 days with a typical diet (51% crude protein and 21 MJ/kg). A total of 400 fish (initial body weight: 54.0 ± 0.0 g) were picked and casually allocated to 16 tanks (25 fish/tank), 4 replicates per dietary treatment. Fish were hand-fed 3 times daily to apparent satiation. The experimental conditions were designed to provide a 10 h light:14 h dark photoperiod (400 lx intensity), aeration was provided continuously in each tank, and water quality parameters were sustained at 24 ± 1 °C, dissolved oxygen > 6.0 mg/L, pH 7.0 to 8.0, and NH_4_–N < 0.3 mg/L.

### 2.4 Sampling procedures

Fish were calculated and weighed at the beginning and end of the experiment. At the beginning of the experiment, 5 fish were casually selected to analyze nutrient composition. At the end of the experiment, after 4 or 24 h starvation, fish were anesthetized with tri-chlorobutanol (250 mg/mL) to obtain a sample.

Following 4 h starvation, fish (5 fish/treatment) were casually selected and sacrificed to obtain the digesta of the distal intestine, which was frozen in liquid nitrogen until the microbial and bile acid profile analysis.

Following 24 h starvation, fish (3 fish/tank) were casually selected, anesthetized, and the body-weight (g), total body-length (cm), viscera weight (g), liver weight (g), and gallbladder weight (g) were individually quantified. These fish were further dissected to obtain the liver, gallbladder, and distal intestine, which were frozen in liquid nitrogen, and persevered at −80 °C for further analysis. Liver and distal intestine samples were collected from 3 fish per tank and stored in 4% paraformaldehyde and acetic acid-methanol-conductivity water (1:6:3, vol:vol:vol) fixative, respectively, for histological examination. Additionally, 2 fish per tank were dissected for liver, gallbladder and distal intestine samples for subsequent bile acid profile analysis (liver and gallbladder) and qPCR gene expression analysis (liver and distal intestine). Plasma from fish (2 fish/tank) was obtained as described by Yu et al. (2019) for biochemical and lipid analysis. Finally, fish (3 fish/tank) were casually selected and stored in a sealable plastic bag at −20 °C for nutrient composition analysis. The leftover fish (11 to 13 per tank) were sacrificed, and the liver was collected for crude lipid analysis, as described in the following section.

### 2.5 Measurement and analytical methods

#### 2.5.1 Biochemical analysis

The AOAC protocol was followed to analyze the composition of each diet and fish sample (Chemists and Horwitz, 1975). Briefly, dry matter was determined in an oven at 105 °C for 24 h until constant weight. Crude ash was determined in a muffle furnace at 550 °C for 16 h. Crude protein and crude lipid were measured using a Kjeltec 2300 Unit (Foss Tecator, Hillerød, Denmark) and a Soxtex System HT Unit (Foss, Hillerød, Denmark), respectively. Gross energy was analyzed by an IKAC2000 Calorimeter (IKA, Staufen, Germany). The amino acid composition of diets was measured at Evonik Industries AG (Beijing, China).

#### 2.5.2 Plasma and liver biochemical assays

The total protein (TP), alanine aminotransferase (ALT), aspartate aminotransferase (AST), alkaline phosphatase (AKP), total cholesterol (TC), total glyceride (TG), high-density lipoprotein-cholesterol (HDL-C), and low-density lipoprotein-cholesterol (LDL-C) in plasma and liver were analyzed by reagent kits (Nanjing Jiancheng Bioengineering Institute; Nanjing, China). The content of non-esterified fatty acid (NEFA) was used the reagent kit (Wako, Japan). All commercial kits were used following the manufacturer's recommendations.

#### 2.5.3 Histological examination

The liver and distal intestine were fixed in paraffin via dehydration and then sliced into 5-μm sections. The sections were stained with hematoxylin and eosin (H & E) and Sirius red staining. The frozen liver sections were fixed in optimal cutting temperature compound via dehydration and sliced into 10-μm sections, which were stained according to the procedures of oil red staining. With Tissue FAXS Imaging Software, each section was automatically captured using a 20 × objective and controlled exposure, motor stage filters, and cameras (PCO, Kehlheim, Germany).

#### 2.5.4 The real-time quantitative PCR analysis

The methods of total RNA extraction, reverse transcription, and real-time quantitative PCR analysis were described by Xie et al. (2022). The database (RNA-seq, accession numbers SRR10158532 and SRR10158533) was used to gain the target genes, and the primer design was described by Yu et al. (2019b). All gene primers are shown in Table S2. The *ef-1α* (accession no. KT827794) was the housekeeping gene for normalization. Each gene generated the amplification efficiency (E-values) from the criterion curve of a serial cDNA sample dilutions ranging from 91.2 to 113.6.

#### 2.5.5 Bile acid profile analysis

Plasma, liver, gallbladder, and distal intestinal chyme samples were prepared following previous reports (Gu et al., 2017; Wei et al., 2021). Then the bile acid profile analysis was performed in UHPLC-TQqQ-MS/MS, and the data was acquired using Agilent Mass Hunter software as described by Wei et al. (2021). There were 19 reference standards of bile acid used in the method, including cholic acid (CA), chenodeoxycholic acid (CDCA), deoxycholic acid (DCA), lithocholic acid (LCA), *α*-muricholic acid (*α* MCA), *β*-muricholic acid (*β* MCA), *ω*-muricholic acid (*ω* MCA), tauro-cholic acid (TCA), tauro-chenodeoxycholic acid (TCDCA), glycol-cholic acid (GCA), glycol-chenodeoxycholic acid (GCDCA), tauro-deoxycholic acid (TDCA), glycol-deoxycholic acid (GDCA), urso-deoxycholic acid (UDCA), glycol-ursodeoxycholic acid (GUDCA), tauro-ursodeoxycholic acid (TUDCA), tauro-*α*-muricholic acid (T*α* MCA), tauro-*β*-muricholic acid (T*β* MCA), and tauro-*ω*-muricholic acid (T*ω* MCA).

#### 2.5.6 Intestinal microbiome analysis

The experimental methods of DNA extraction, PCR amplification, and PCR production purification are described by Xie et al. (2022). The 16s rRNA genes' V3–V4 hypervariable region primers were 336F: 5′-GTACTCCTACGGGAGGCAGCA-3′ and 806R: 5′-GTGGACTACHVGGGTWTCT-AAT-3'. The sequence of production and bioinformatics analysis were performed at Majorbio Bio-Pharm Technology Co. Ltd. (Shanghai, China) as follows. The purified products were profiled in equimolar and paired-end sequences on an Illumina MiSeq PE300 platform/NovaSeq PE250 platform (Illumina, San Diego, United States). Then, the raw 16S rRNA sequences were analyzed using fastp (Chen et al., 2018), FLASH (Magoč and Salzberg, 2011), QIIME2 (Bolyen et al., 2019; Callahan et al., 2016), and SILVA 16S rRNA database (v13.8) to obtain the high-quality sequences on the Majorbio Website (cloud.majorbio.com). Then, using the heatmap package of R (version 3.3.1), we analyzed the Spearman's rank correlation, and TBtools software (Chen et al., 2020a) was used to visualize the correlation graph.

### 2.6 Calculations

Different bile acid concentrations were calculated based on (Chen et al., 2020b; Gu et al., 1992): (1) Total bile acids = sum of all bile acid concentrations, (2) total primary bile acids = (CA + TCA + GCA + CDCA + TCDCA + GCDCA), (3) total secondary bile acids = (DCA + TDCA + GDCA + LCA + UDCA + TUDCA + GUDCA + *α* MCA + T*α* MCA + *β* MCA + T*β* MCA + *ω* MCA + T*ω* MCA), (4) conjugated bile acids = (T*α* MCA + T*β* MCA + T*ω* MCA + GCDCA + TCDCA + GDCA + TDCA + GUDCA + TUDCA); (5) unconjugated bile acids = (*α* MCA + *β* MCA + *ω* MCA +CDCA + CA + GCA + TCA + UDCA + DCA + LCA).

### 2.7 Statistical analysis

All data are presented as mean ± standard deviation. The normality and homogeneity of variance on all data was analyzed using the typical QQ plot and Shapiro–Wilk test, except for histologic, microbiome and bile acid data. The data were transformed as needed into a normal distribution and analyzed via one-way ANOVA in SPSS 22.2. The histologic data were analyzed using the Chisq. post.hoc test. The microbiome and bile acid data were analyzed using the Wilcoxon/Kruskal–Wallis test since these did not meet the standard distribution requirement. GraphPad software (USA) was used to draw the data graph.

## 3 Result

### 3.1 Growth, body indices and whole-body composition

The lowest survival rate (48%) was found in the SPC group, while other treatments ranged from 97% to 100% (Table 2). Fish fed the SPC, CPC, and CPCY diets showed lower growth performance as shown in final body weight, weight gain rate, and specific growth rate, lower feed intake compared with FM (Table 2, *P* < 0.05). Only SPC group showed a higher feed conversion ratio compared with other groups (Table 2, *P* < 0.05). However, these performance parameters showed no significant differences between CPC and CPCY (Table 2, *P* > 0.05). And there were no significant diet effects on viscera somatic index and hepatosomatic index (Table 2, *P* > 0.05), but fish fed the SPC, CPC, and CPCY diets had significantly lower gallbladder-somatic index (GBSI) compared with FM (Table 2, *P* < 0.05). Finally, K was significantly lower in the CPC group but not in the SPC and CPCY groups compared with FM (Table 2). Thus, we therefore chose to omit this group in the additional analyses due to the relatively low survival rate in the SPC.

**Table 2:** Growth and feed performance of largemouth bass among dietary treatments.

Item	Diet^1^			
	FM	SPC	CPC	CPCY
**Growth and feed performance**				
Survival^2^, %	100 ± 0^a^	48 ± 8^b^	98 ± 2^a^	97 ± 4^a^
BWi, g/fish	54.0 ± 0.0	54.0 ± 0.0	54.0 ± 0.0	54.0 ± 0.0
BWf, g/fish	193.2 ± 5.4^a^	165.1 ± 7.7^b^	165.4 ± 7.6^b^	162.1 ± 4.9^b^
FI, g/tank	3746 ± 74^a^	2194 ± 158^c^	3161 ± 96^b^	2987 ± 63^b^
WGR^3^, %	257.6 ± 9.9^a^	48.1 ± 19.1^c^	200.2 ± 14.9^b^	191.2 ± 17.7^b^
SGR^4^, %/day	1.82 ± 0.04^a^	1.59 ± 0.13^b^	1.60 ± 0.07^b^	1.57 ± 0.04^b^
FCR^5^	1.08 ± 0.00^b^	5.35 ± 4.08^a^	1.17 ± 0.03^b^	1.16 ± 0.09^b^
**Body indices**				
K^6^	1.93 ± 0.17^a^	1.86 ± 0.11^ab^	1.76 ± 0.06^b^	1.88 ± 0.15^a^
VSI^7^, %	7.08 ± 0.92	6.67 ± 1.07	6.75 ± 0.71	7.18 ± 0.57
HSI^8^, %	1.54 ± 0.51	1.32 ± 0.52	1.23 ± 0.21	1.39 ± 0.16
GBSI^9^, %	0.11 ± 0.01^a^	0.08 ± 0.01^b^	0.09 ± 0.01^b^	0.08 ± 0.01^b^
**Whole-body, % wet weight**				
Crude protein	17.5 ± 0.1	–	17.3 ± 0.1	17.2 ± 0.1
Crude lipid	9.7 ± 0.4^a^	–	9.0 ± 0.2^b^	8.8 ± 0.4^b^
Crude ash	3.9 ± 0.1	–	4.0 ± 0.0	3.9 ± 0.0

BWi = initial body weight; BWf = final body weight; FI = apparent feed intake; WGR = weight gain rate; SGR = specific growth rate; FCR = feed conversion ratio; VSI = viscera somatic index; HSI = hepatosomatic index; GBSI = gallbladder-somatic index. ^a-c^Within a row, means without a common superscript differ significantly (Duncan's test; *P* < 0.05, mean ± standard deviation, *n* = 4 for growth and feed performance, and whole-body parameters; *n* = 12 for body indices).

1 FM = fish meal diet; SPC = soy protein concentrate diet; CPC = cottonseed protein concentrate diet; CPCY = CPC diet +800 mg/kg multi-strain yeast fractions (MsYF).

2 Survival = 100 × final fish number/initial fish number.

3 WGR (%) = 100 × [(BMf − BMi)/BMi], where BMi is the total initial fish biomass.

4 SGR (%/day) = 100 × [ln (BWf) – ln (BWi)]/*n*, where *n* = 70.

5 FCR = FI/(BWf − BWi).

6 K = 100 × BW/TL^3^, where BW is the body weight in grams, and TL is the total body length in centimeters.

7 VSI (%) = 100 × VW/BW, where VW is the viscera weight and BW is body weight, both in grams.

8 HSI (%) = 100 × LW/BW, where LW is the liver weight and BW is body weight, both in grams.

9 GBSI (%) = 100 × GBW/BW, where GBW is the gallbladder weight and BW is body weight, both in grams.

In the whole-body indices, there were no statistical changes in the crude protein and ash among diets (Table 2, *P* > 0.05). In contrast, the crude lipid level was significantly lower in CPC and CPCY than in FM (Table 2, *P* < 0.05), but no differences were found between CPC and CPCY (Table 2, *P* > 0.05).

### 3.2 Histopathological and hematological liver functions

**Fig. 1:**
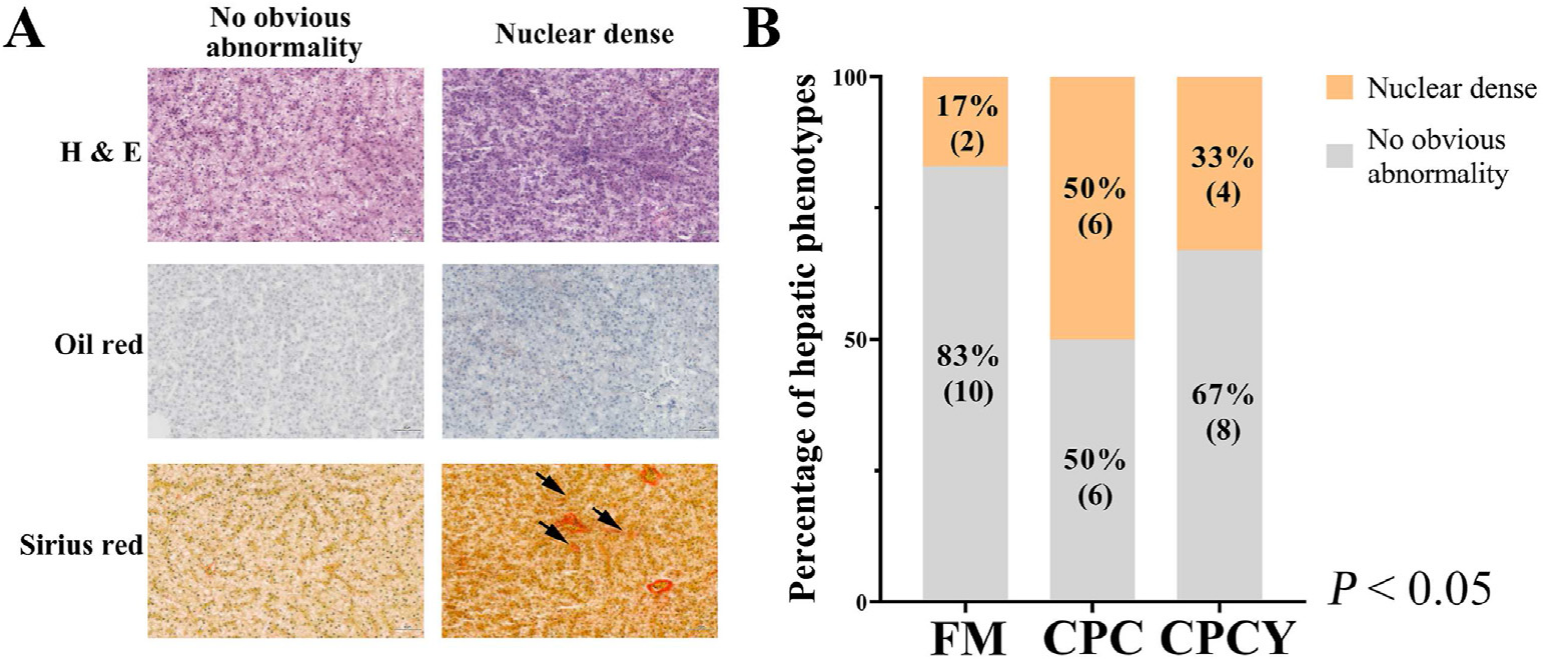
Histopathological examination of largemouth bass among dietary treatments. (A) Hematoxylin and eosin (H & E), oil red and Sirius red staining (the red collagen fibers marked by black arrow) of liver; (B) Contingency chart showing proportions of 2 hepatic phenotypes. *P*-values of the Fisher's exact test are shown on the lower right corner of the subplot. FM = fish meal diet; CPC = cottonseed protein concentrate diet; CPCY = CPC diet +800 mg/kg multi-strain yeast fractions (MsYF).

There were 2 phenotypes of liver observed in all treatments, as reported by Chen et al. (2022). The phenotype with no obvious abnormality showed well-shaped cells with evenly dispersed cytoplasm and fewer collagen fibers (Fig. 1A). Compared to no obvious abnormality, the hepatic disease-nuclear dense phenotype showed higher nucleus density, abnormal hepatocytes characterized by unclear liver cord and increased collagen fibers (Fig. 1A). Based on the Oil red staining, no clear lipid accumulation signal was observed in both phenotypes (Fig. 1A). As shown in Fig. 1B, fish fed CPC diet showed a high rate of hepatic disease (50%) compared to those fed FM diet (17%). In comparison, the MsYF supplementation (CPCY diet) reduced the proportion of fish presenting a nuclear dense phenotype to 33%. There were no obvious phenotypic abnormalities in distal intestinal samples among treatments shown in Fig. S1. Meanwhile, compared with FM, fish fed CPC diet showed lower AKP but higher TP, ALT, and AST levels (Table 3, *P* < 0.05), and these plasma biochemical parameters showed no significant differences between CPC and CPCY (Table 3, *P* > 0.05).

**Table 3:** Plasma biochemical parameters of largemouth bass among dietary treatments.

Item	Diet^1^		
	FM	CPC	CPCY
TP, mmol/L	15.67 ± 1.62^b^	18.01 ± 1.79^a^	18.89 ± 1.99^a^
ALT, U/L	4.72 ± 1.79^b^	6.18 ± 1.14^a^	5.76 ± 0.97^ab^
AST, U/L	0.85 ± 0.43^b^	2.47 ± 1.21^a^	1.76 ± 0.51^a^
AKP, U/L	47.68 ± 10.73^a^	22.94 ± 11.20^b^	28.42 ± 9.72^b^

TP = total protein; ALT = alanine aminotransferase; AST = aspartate aminotransferase; AKP = alkaline phosphatase.

1 FM = fish meal diet; CPC = cottonseed protein concentrate diet; CPCY = CPC diet +800 mg/kg multi-strain yeast fractions (MsYF). ^a, b^ Within a row, means without a common superscript differ significantly (Duncan's test; *P* < 0.05, mean ± standard deviation, *n* = 8).

### 3.3 Lipid metabolism

**Fig. 2:**
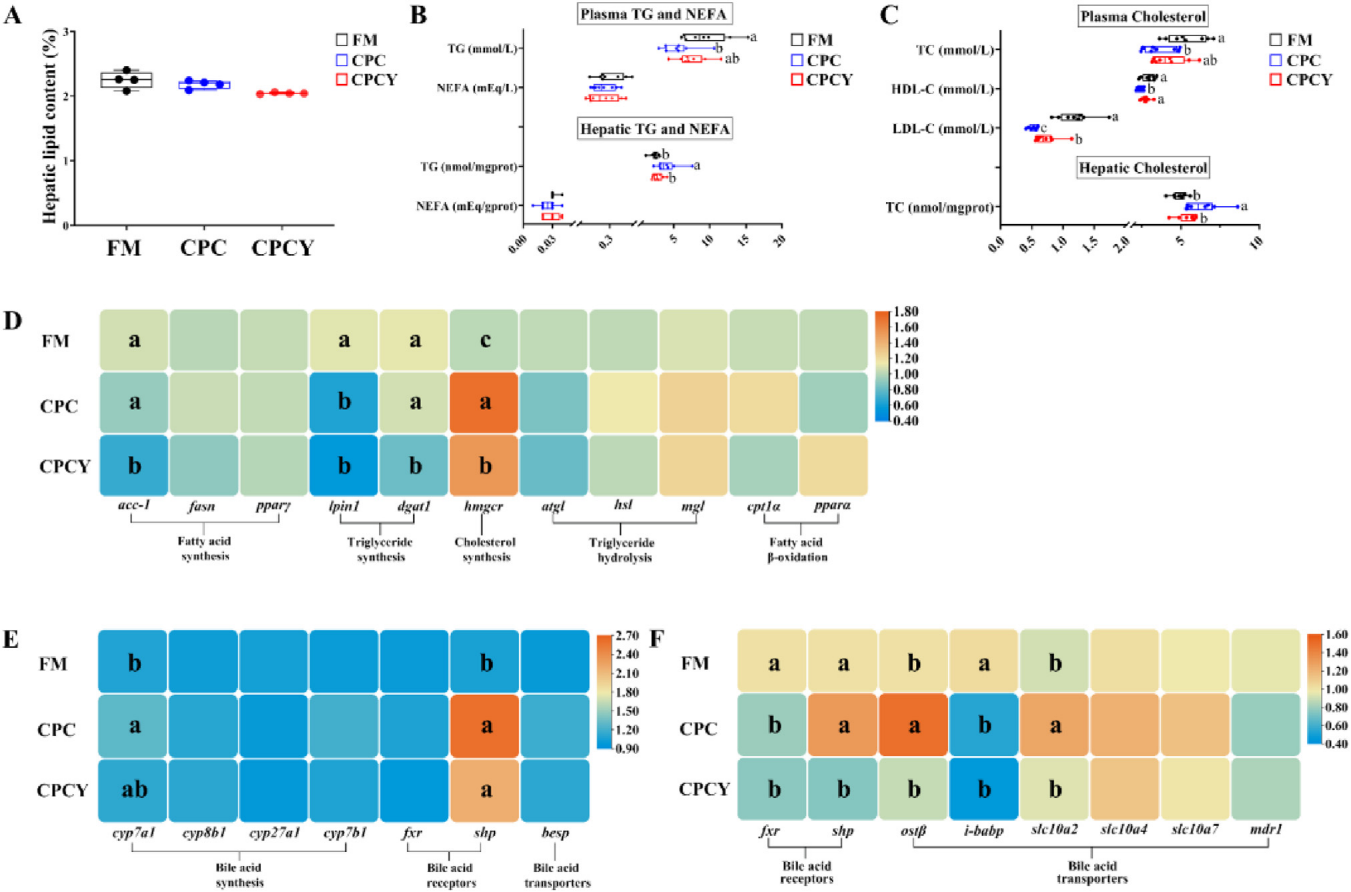
Lipid metabolism of largemouth bass fed experimental diets during the 10-week experiment. (A) Hepatic lipid content (*n* = 4). (B) Plasma and hepatic total glyceride (TG) and non-esterified fatty acid (NEFA) (*n* = 8). (C) Plasma and hepatic total cholesterol (TC) (*n* = 8). (D) Hepatic lipogenesis (*acc-1* = acetyl CoA carboxylase-1; *fasn* = fatty acid synthase; *pparγ* = peroxisome proliferators-activated receptors γ; *lpin1* = lipid phosphate phosphohydrolase 1; *dgat1* = diacylglycerol acyltransferase 1; *hmgcr* = 3-hydroxy-3-methyl glutaryl coenzyme A reductase) and lipolysis (*atgl* = adipose triglyceride lipase; *hsl* = hormone-sensitive lipase; *mgl* = monoacylglycerol lipase; *cpt1α* = carnitine palmitoyltransferase 1α; *pparα* = peroxisome proliferators-activated receptors α) gene expression levels (*n* = 8); (E) Hepatic bile acid metabolism (*cyp7a1* = cytochrome P450 7A1; *cyp8b1* = cytochrome P450 8B1; *cyp27a1* = cytochrome P450 27A1; *cyp7b1* = cytochrome P450 7B1; *fxr* = farnesoid X receptor; *shp* = nuclear receptor subfamily 0 group B member 2; *besp* = bile salt export pump) gene expression levels (*n* = 8); (F) Distal intestinal bile acid metabolism (*fxr* = farnesoid X receptor; *shp* = *nuclear receptor subfamily 0 group B member 2*; *ostβ* = organic solute transporter β; *i-babp* = ileal bile acid binding protein; *slc10a2* = sodium/bile acid cotransporter 2; *slc10a4* = sodium/bile acid cotransporter 4; *slc10a7* = sodium/bile acid cotransporter 7; *mdr1* = multidrug resistance protein 1) gene expression levels (*n* = 8). ^a-c^Means without a common superscript differ significantly (Duncan's test; *P* *<* 0.05). Data are shown as mean ± standard deviation. FM = fish meal diet; CPC = cottonseed protein concentrate diet; CPCY = CPC diet + 800 mg/kg multi-strain yeast fractions (MsYF).

There were no statistical changes in the hepatic lipid content and NEFA content of plasma and liver among diets (Fig. 2A and B, *P* > 0.05). Fish fed the CPC diet reduced plasma TG, TC, HDL-C, and LDL-C levels, and increased TG and TC contents in the liver compared to those fed the FM diet (Fig. 2B–C, *P* < 0.05). Regarding the effect of MsYF, fish fed the CPCY diet showed no significant differences in the levels of plasma TG and plasma TC, but showed significant increases of plasma HDL-C and LDL-C levels compared to those fed the CPC diet (Fig. 2B–C). Meanwhile, fish fed the CPCY diet had significantly lower levels of liver TG and liver TC content than those fed the CPC diet (Fig. 2B–C, *P* < 0.05).

In the CPC and CPCY groups, fish showed higher gene expression levels related to cholesterol synthesis, i.e., 3-hydroxy-3-methyl glutaryl coenzyme A reductase (*hmgcr*), and lower expression of triglyceride synthesis, i.e., lipid phosphate phosphohydrolase 1 (*lpin1*), compared to those fed the FM diet (Fig. 2D, *P* < 0.05). For the effect of MsYF, compared with CPC, fish fed the CPCY diet had lower gene expression of acetyl CoA carboxylase-1 (*acc-1*), diacylglycerol acyltransferase 1 *(dgat1*), and *hmgcr* expression levels (Fig. 2D, *P* < 0.05). Other genes related to lipid synthesis (fatty acid synthase [*fasn*]; peroxisome proliferators-activated receptors [*pparγ*]) and lipolysis (adipose triglyceride lipase [*atgl*]; hormone-sensitive lipase [*hsl*]; monoacylglycerol lipase [*mgl*]; carnitine palmitoyltransferase 1α [*cpt1α*]; peroxisome proliferators-activated receptors α [*pparα*]) were not significantly changed by diets (Fig. 2D, *P* > 0.05).

### 3.4 Bile acid metabolism

Dietary CPC showed higher expression of cytochrome P450 7A1 (*cyp7a1*) and nuclear receptor subfamily 0 group B member 2 (*shp*) than those fed the FM diet in the liver (Fig. 2E, *P* < 0.05). Dietary CPC significantly reduced the expression of farnesoid X receptor (*fxr*) and ileal bile acid binding protein (*i-babp*), but increased that of organic solute transporter β (*ostβ*) and sodium/bile acid cotransporter 2 (*slc10a2*) in the distal intestine (Fig. 2F, *P* < 0.05). Regarding the MsYF, fish fed CPCY significantly down-regulated the expression levels of *shp*, *ostβ,* and *slc10a2* in the distal intestine compared with CPC (Fig. 2F, *P* < 0.05).

The expression of other bile acid-relevant genes in the liver, including cytochrome P450 8B1 (*cyp8b1*), cytochrome P450 27A1 (*cyp27a1*), cytochrome P450 7B1 (*cyp7b1*), *fxr*, and bile salt export pump (*besp*), were not significantly influenced by dietary treatments (Fig. 2E, *P* > 0.05). And the expression levels of bile acid transporters (sodium/bile acid cotransporter 4 [*slc10a4*]; sodium/bile acid cotransporter 7 [*slc10a7*]; multidrug resistance protein 1 [*mdr1*]) in distal intestine were not significantly influenced by dietary treatments (Fig. 2F, *P* > 0.05).

### 3.5 Bile acid profile

**Fig. 3:**
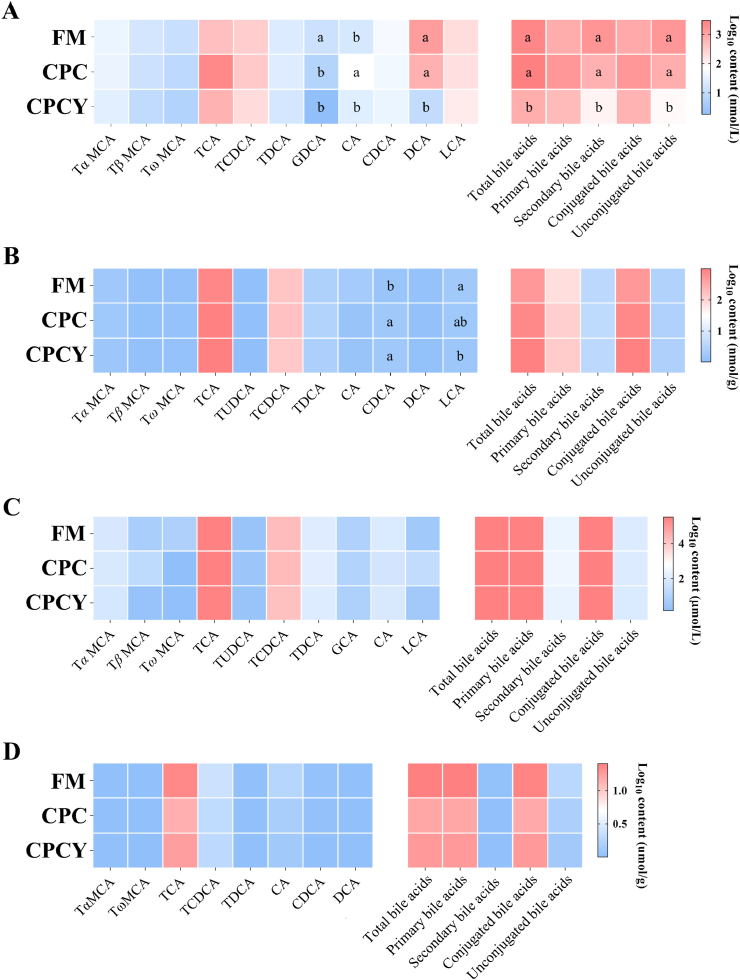
Bile acids profile in different tissues of largemouth bass among dietary treatments. (A) Plasma. (B) Liver. (C) Gallbladder. (D) Distal intestinal chyme. ^a,b^ Means without a common superscript differ significantly (Wilcoxon/Kruskal–Wallis test; *P <* 0.05, *n* = 8 for plasma, liver, and gallbladder data; *n* = 5 for distal intestinal chyme data). Data are shown as the mean. FM = fish meal diet; CPC = cottonseed protein concentrate diet; CPCY = CPC diet +800 mg/kg multi-strain yeast fractions (MsYF). T*α* MCA = tauro-*α*-muricholic acid; T*β* MCA = tauro-*β*-muricholic acid; T*ω* MCA = tauro-*ω*-muricholic acid; TCA = tauro-cholic acid; TCDCA = tauro-chenodeoxycholic acid; TUDCA = tauro-ursodeoxycholic acid); TDCA = tauro-deoxycholic acid; GCA = glycol-cholic acid; GDCA = glycol-deoxycholic acid; CA = cholic acid; CDCA = chenodeoxycholic acid; DCA = deoxycholic acid; LCA = lithocholic acid.

In Fig. S2, the bile acid profile of plasma, liver, gallbladder and distal intestinal chyme identified the following: TCA, DCA, TCDCA, LCA, CDCA, T*α* MCA, TDCA, CA, T*β* MCA, GDCA, TUDCA, and T*ω* MCA. Compared to the FM group, dietary CPC significantly increased CA content and decreased GDCA content in the plasma (Fig. 3A, *P* < 0.05). Regarding the liver bile acid profile, dietary CPC showed higher CDCA content than FM (Fig. 3B, *P* < 0.05). Regarding the effect of MsYF, it significantly decreased plasma levels of CA, DCA, total bile acids, secondary bile acids, and unconjugated bile acids compared with CPC (Fig. 3A, *P* < 0.05). Further, dietary MsYF significantly reduced the LCA content in the liver compared with FM (Fig. 3B, *P* < 0.05). Dietary treatments influenced the bile acid composition in the gallbladder and distal intestinal chyme marginally (Fig. 3C–D, *P* > 0.05).

### 3.6 Gut microbiota analysis

**Fig. 4:**
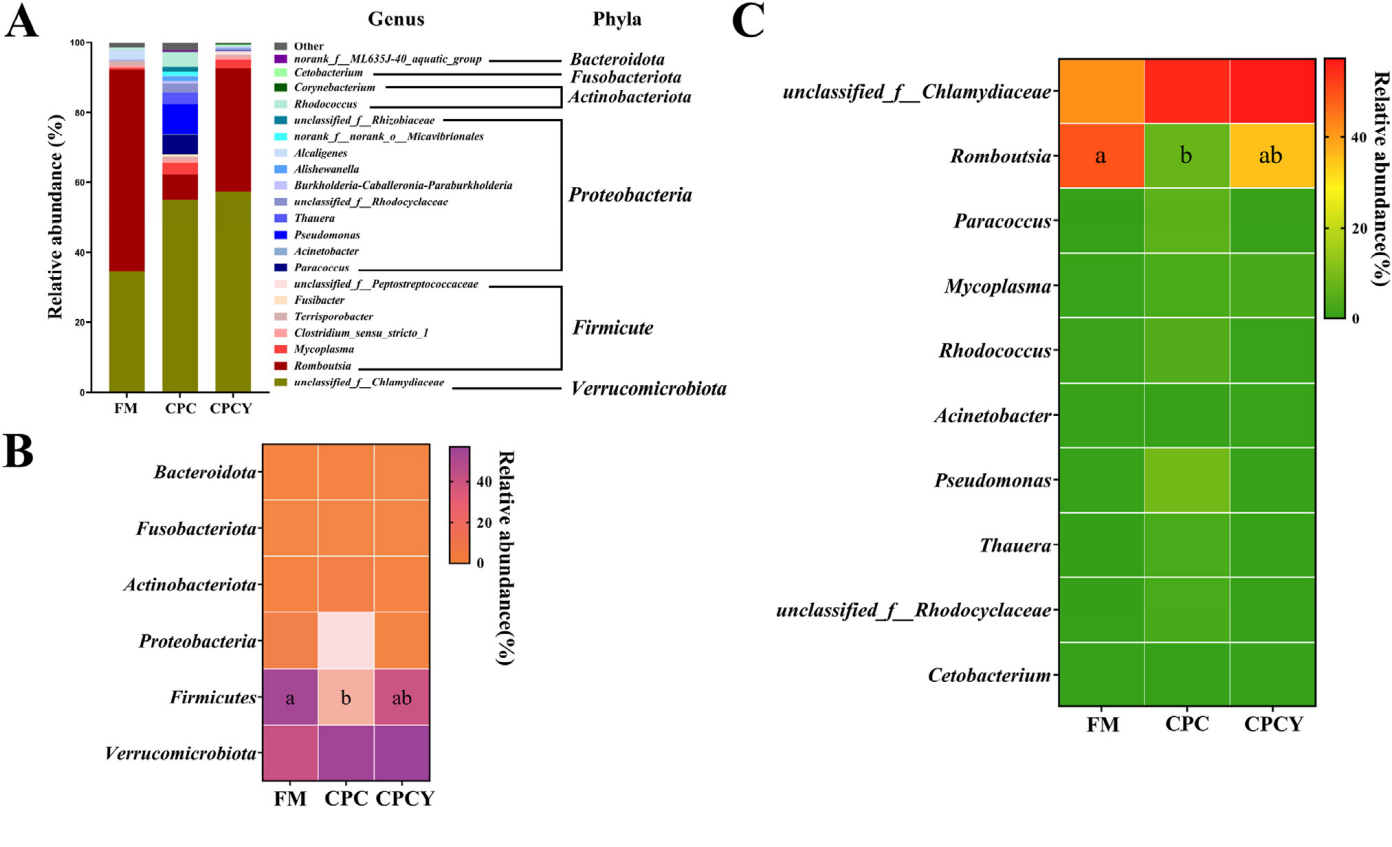
Gut microbiota composition of distal intestine of largemouth bass. (A) The relative abundance of each taxon among dietary treatments. (B) The top 6 most abundant phyla among dietary treatments. (C) The top 10 most abundant genera among dietary treatments. ^a,b^ Means without a common superscript differ significantly (Wilcoxon/Kruskal–Wallis test; *P* < 0.05, *n* = 5). Data are shown as the mean. FM = fish meal diet; CPC = cottonseed protein concentrate diet; CPCY = CPC diet + 800 mg/kg multi-strain yeast fractions (MsYF).

At phylum level, the phyla Verrucomicrobiota, Firmicutes, and Proteobacteria were the top 3 abundant phyla among treatments (Fig. 4A and B). The abundance of Firmicutes in CPC was decreased compared to FM (Fig. 4B, *P* < 0.05), while dietary MsYF reversed its abundance to the levels observed in FM (Fig. 4B, *P* > 0.05). At genus level, the top 10 genera are shown in Fig. 4C. The *unclassified_f__*Chlamydiaceae was the dominant bacterium across dietary groups accounting for 41% to 57% (Fig. 4C). Compared with FM, dietary CPC significantly reduced the abundance of genus *Romboutsia* (Fig. 4C, *P* < 0.05), while such change was not observed in fish fed the diet supplemented with MsYF, i.e., CPCY diet (Fig. 4C, *P* > 0.05).

**Fig. 5:**
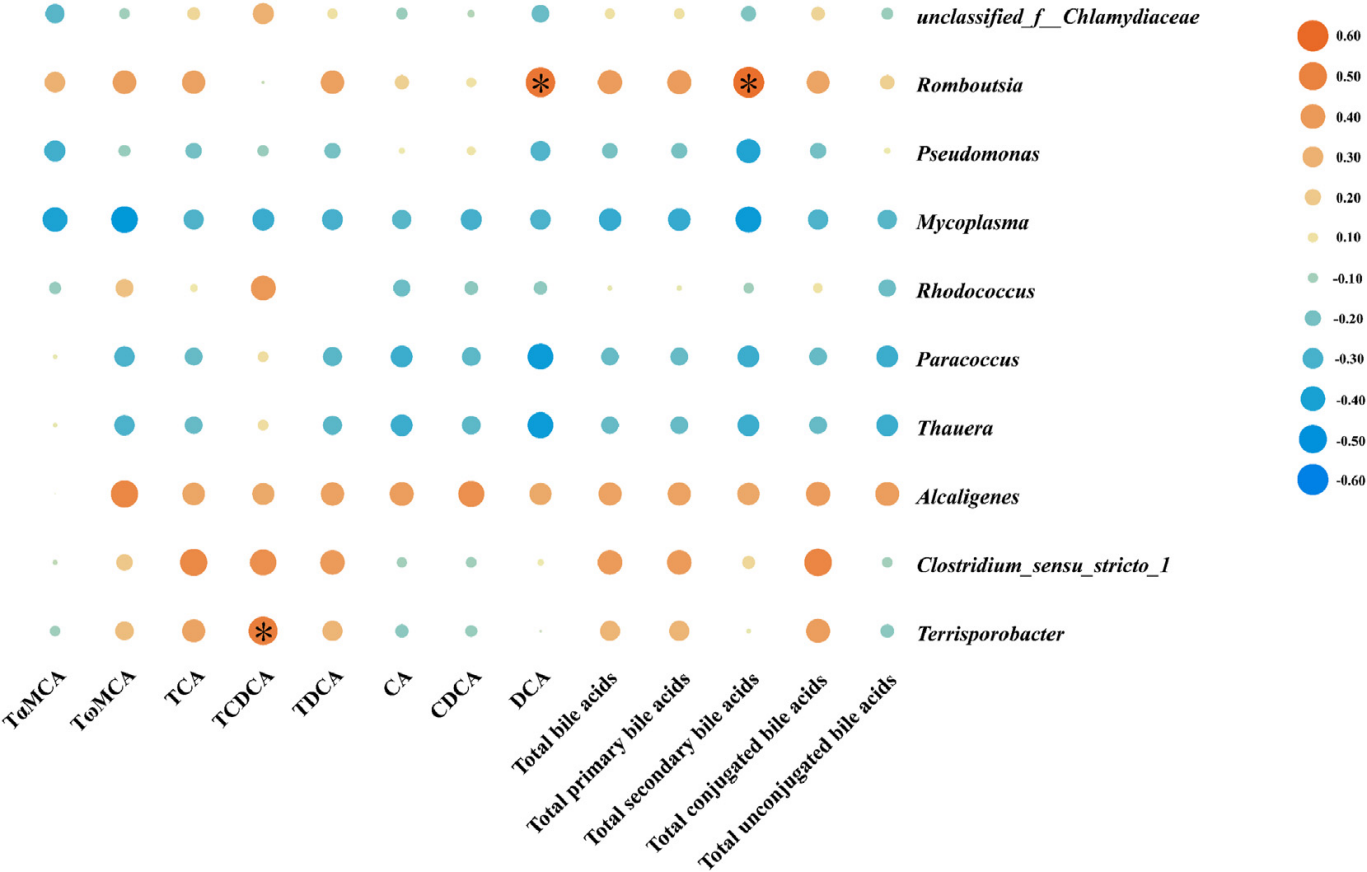
The Spearman correlation between gut microbiota and distal intestinal chyme bile acids profile. The gradient colors of circles represent the correlation coefficients, with red indicating more positive, and blue indicating more negative. Values marked with a black symbol “∗” are significant correlations (*P* < 0.05). T*α* MCA = tauro-*α*-muricholic acid; T*ω* MCA = tauro-*ω*-muricholic acid; TCA = tauro-cholic acid; TCDCA = tauro-chenodeoxycholic acid; TDCA = tauro-deoxycholic acid; CA = cholic acid; CDCA = chenodeoxycholic acid; DCA = deoxycholic acid.

According to the Spearman correlation analysis, the contents of total secondary bile acids and DCA of distal intestine chyme had a positive correlation with genus *Romboutsia*, and TCDCA had a positive correlation with genus *Terrisporobacter* (Fig. 5, *P* < 0.05).

## 4 Discussion

### 4.1 CPC affects the growth and lipid metabolism in largemouth bass

In this study, the fishmeal substitution level by soy protein concentrate or cottonseed protein concentrate was increased to 81%, which was found to significantly compromise growth and feed performance. Largemouth bass fed SPC diet showed the lowest survival (48%) among treatments indicating that fish might not tolerate this substitution level of soy protein concentrate. Carral et al. (2021) have reported that the increasing incorporation level of soy protein concentrate (45% to 100% fishmeal substitution) led to an increment of ANFs, like trypsin inhibitor and glycinin antigen, resulting in enteritis and poor growth performance of *Tinca tinca* L. (Carral et al., 2021), as supported by our findings. In contrast, the survival of CPC and CPCY ranged from 97% to 98%, indicating that largemouth bass might tolerate this substitution level of cottonseed protein concentrate. Of note, the growth performance was still suppressed in cottonseed protein concentrate-based diets compared to those fed the FM diet, which was in agreement with other studies (Liu et al., 2021; Mohd Faudzi et al., 2018). Compared to FM, the lower feed intake in the CPC-containing treatments during the experimental period indicated poor palatability in largemouth bass, which could cause poor growth (Liang et al., 2019; Nagel et al., 2012). Additionally, the crude cellulose and non-starch polysaccharide contents in the CPC diet were higher than those in the FM diet, which may disturb the nutrient digestibility (Francis et al., 2001), and then reduce the growth. However, in the same species, Wang and co-authors have reported that the level of CPC substitution could be up to 80% of fishmeal protein without a negative effect on fish growth (Wang et al., 2021b). These inconsistent results might be due to the differences in fish genetics, body size, farming conditions, etc (Biswas et al., 2019).

Compared to FM, fish fed the CPC diet showed a higher rate of hepatic disease, which was consistent with one study that the 75% cottonseed protein concentrate substitution level could induce liver disease in largemouth bass (Liu et al., 2021). The increased hepatic TC and TG levels alongside up-regulated cholesterol synthesis genes, i.e., *hmgcr*, could be responsible for the hepatic disease in CPC as the excess hepatic TC and TG accumulation was one of the inducible factors of liver damage in largemouth bass (Chen et al., 2022). Further, the increment of plasma AST and ALT in CPC supported liver damage in this group. Similarly, in hybrid grouper, cottonseed protein concentrate-containing diets (36% to 48% fishmeal substitution) induced not only a severe fat deposition in the liver but also an increase of plasma AST and ALT levels (Yin et al., 2018). Furthermore, as 2 crucial cholesterol transporters, the decrease of plasma HDL-C and LDL-C in CPC also indicated a lower ability to carry cholesterol from peripheral tissues to liver, which was in agreement with prior findings in *Scophthalmus maximus****,*** fed a plant-based diet (wheat gluten meal:soybean meal = 3.7:8.4, 75% fishmeal substitution) (Yun et al., 2011). Interestingly, the down-regulation of triglyceride synthesis associated gene (*lpin1*) in CPC seemed to be contrary to the increment of hepatic TG. It is unknown but highly likely that the down-regulation of *lpin1* drastically reduced LDL-TG secretion, and then increased the hepatic TG level (Finck, 2010).

The conversion of cholesterol into bile acid is vital to prevent the accumulation of TC in the liver (Russell, 1992). Our study showed that fish fed the CPC diet had a smaller GBSI suggesting insufficient bile acid secretion resulting in the reduction of lipid absorption from diets and even a decrement in the crude lipid of whole fish composition, as supported by our findings. Similarly, rainbow trout fed a soybean diet showed smaller GBSI and lower lipid digestibility, suggesting insufficient bile acid secretion (Yamamoto et al., 2010). Also, insufficient bile acid secretion might stimulate the up-regulation of bile acid synthesis (*cyp7a1*) in largemouth bass fed the CPC diet via negative feedback regulation in the liver (Zhou and Hylemon, 2014). However, as a carnivorous fish, largemouth bass fed the plant-based diet showed inhibition of bile acid synthesis (*cyp7a1*) even though the gene expression of TC synthesis (*hmgcr*) was high (Yu et al., 2019a). A similar study in Japanese seabass found a plant-based diet (soy protein concentrate:cottonseed protein concentrate = 2.3:3.82; 100% fishmeal substitution) induced the hyper-synthesis of TC and lower synthesis of bile acid resulting in the accumulation of hepatic TC (Zhang et al., 2019). The mechanisms behind these inconsistent results remain unknown and need further investigation. Notably, we found that dietary CPC down-regulated the expression level of bile acid receptor (*fxr*) and transporter (*i-babp*) in the distal intestine, which might imply a disturbance in bile acid transport. However, the up-regulation of intestinal *slc10a2* expression level seemed to accelerate bile acid transport from the gallbladder to the intestine (Oelkers et al., 1997). However, the down-regulation of *i-babp* reduced the bile acid binding capacity leading to a delay in intracellular bile acid transport (Mosińska et al., 2018). Mosińska and co-authors found that, in the intestine, *fxr* mediated the *i-babp* expression level to increase the bile acid binding capacity for accelerating intracellular bile acid transport (Mosińska et al., 2018). Wei et al. (2019) found that enhancement of hepatic bile acid synthesis could increase the micro-vascularization ratio of the liver in amur sturgeon, *Acipenser schrenckii*, fed a whole plant protein diet (soy protein concentrate:cottonseed protein concentrate = 2.30:4.08; 100% fishmeal substitution) (Wei et al., 2020); which was similar to our findings that a higher proportion of fish had a higher rate of hepatic disease in the CPC group. Wei and co-authors suggested that a plant-based diet could damage intestinal morphology and then decrease intestinal bile acid transport (Wei et al., 2020). In our study, a high cottonseed protein concentrate substitution level did not appear to damage the morphology of distal intestine, although distal intestinal bile acid transport was disturbed. It is highly likely that certain micronutrients, such as taurine, functional peptides, and unknown growth factors, are relatively low in CPC diets (Sampath et al., 2020). Thus, the dysfunction of intestinal bile acid transport and increase in bile acid synthesis contributed to a bile acid disturbance in fish fed the CPC diet, i.e., the increment of plasma CA and hepatic CDCA. As the usual bile acids, CA and CDCA are the signal stimulators (Chiang and Ferrell, 2019), whose over-accumulation could activate inflammation-related signaling, like ROS and NLRP3 signaling, which intensify the inflammation response and damage to hepatic cells (Gong et al., 2016; Yamada et al., 2017). For largemouth bass, how the increment in the content of these bile acids affects liver health needs further investigation.

Regarding the gut microbiota, dietary CPC reduced the genus *Romboutsia* abundance which showed a positive correlation with the total secondary bile acids (DCA). One recent study revealed that the species *Romboutsia ilealis* could have the potential genic function of expressing bile salt hydrolase (Gerritsen et al., 2017) suggesting that a lower relative abundance of genus *Romboutsia* might slow down intestinal bile acid hydrolysis and in turn influence the bile acid profile in fish fed the CPC diet. Of note, the characterization of the associations between physiological functions and intestinal microbial clades is a fundamental method of finding the physiological functions of bacteria, which could benefit fish health and welfare (Guo et al., 2022; Wang et al., 2022b; Wang et al., 2021a; Wang et al., 2022c). However, studies on the correlation between *Romboutsia* and bile acid metabolism in fish are still rare and need further investigation.

### 4.2 MsYF mitigate the hepatic lipid accumulation and alter bile acid metabolism

Dietary MsYF could improve the growth of fish via the amelioration of tissue health (Rawling et al., 2019; Xie et al., 2022). In this study, MsYF supplementation clearly decreased the hepatic disease proportion of fish, indicating improved liver health. Similarly, the beneficial effects of MsYF on intestinal health have been observed in rainbow trout via the stimulation of mucosal immune response (Rawling et al., 2021). As mentioned above, the accumulation of hepatic TG and TC induced by dietary CPC might indicate an abnormal liver. With MsYF supplementation, the hepatic TG and TC were clearly decreased, accompanied by the decreasing appearance of lipid accumulation. Additionally, fish fed MsYF increased plasma HDL-C and LDL-C, suggesting the improvement in lipid transporter ability. High plasma HDL-C in largemouth bass suggested lipid transport acceleration preventing excessive hepatic lipid accumulation (Yu et al., 2019b). Further, dietary MsYF significantly down-regulated the expression of some lipid synthesis genes, like *acc-1*, *dgat1*, and *hmgcr*, which were consistent with some studies that yeast products could reduce lipid accumulation via regulation of lipid metabolism in the liver (Cao et al., 2016; Yu et al., 2019b).

In terms of bile acid metabolism, compared to fish fed a high level of cottonseed protein concentrate without paraprobiotics, dietary MsYF clearly reduced the plasma total bile acid contents suggesting an improvement in host health. This finding could be attributed to bile acid transport, as reflected in the increased gene expression of the bile acid transporters (*ostβ* and *slc10a2*). Both *ostβ* and *slc10a2* play an essential role in bile acid transport, especially the CA, CDCA, DCA, and LCA (Ballatori et al., 2009; Oelkers et al., 1997), suggesting up-regulation of *ostβ* and *slc10a2* might influence the plasma bile acid profile, as supported by our findings, while dietary MsYF could regulate bile acid transporters, and decrease the plasma total bile acid level. Additionally, in agreement with the previous study (Xie et al., 2022), dietary MsYF altered the microbiome composition towards a gut microbial composition more similar to that of the FM group, remarkably increasing the abundance of genus *Romboutsia*. Gerritsen (2015) reported that *Romboutsia* has bile acid-hydrolysis and short-chain fatty acid production abilities. An increase in its abundance might accelerate the synthesis of secondary bile acids and short-chain fatty acids, partly supported by our finding that the total secondary bile acids (DCA) in distal intestine chyme positively correlated with the genus *Romboutsia* (Gerritsen, 2015). However, dietary MsYF did not significantly change the bile acid profile of distal intestine chyme. The potential mechanisms behind these findings remain unknown and need further investigation.

## 5 Conclusion

Compared to cottonseed protein concentrate, soy protein concentrate that replaced 81% fishmeal protein strictly suppressed the survival and growth of largemouth bass. Largemouth bass fed the 81% fishmeal protein substitution by cottonseed protein concentrate did not significantly suppress the survival but induced higher hepatic lipid accumulation and disturbance in bile acid and microbiota profile, which were concomitant with a reduction in growth and feed performance. In addition, the high cottonseed protein concentrate level in the diet caused the dysbiosis of the gut microbiome characterized, in particular, by a reduced prevalence of the potentially beneficial genus *Romboutsia*.

Regarding the effect of dietary paraprobiotics, fish fed CPCY mitigated hepatic lipid accumulation, reduced bile acid transport and total plasma bile acids levels, as well as improved liver function and supported maintenance of microbiota composition. Together, these findings provide a positive contribution to support the substitution of fishmeal with cottonseed protein concentrate in the diet of largemouth bass.

## Author contributions

**Xiaoze Xie**: Conceptualization, Methodology, Investigation, Formal analysis, Writing - Original Draft. **Xiaofang Liang**: Methodology, Formal analysis. **Hao Wang**: Methodology. **Qiang Zhu**: Software. **Junjun Wang**: Validation. **Ying Chang**: Resources. **Eric Leclercq**: Project administration, Writing - Review & Editing. **Min Xue**: Project administration, Resources. **Jie Wang**: Supervision, Formal analysis, Writing - Review & Editing.

## Declaration of competing interest

We declare that we have no financial and personal relationships with other people or organizations that can inappropriately influence our work, and there is no professional or other personal interest of any nature or kind in any product, service and/or company that could be construed as influencing the content of this paper.
