# Supplementary material for: Effects of paraprobiotics on bile acid metabolism and liver health in largemouth bass (Micropterus salmoides) fed a cottonseed protein concentrate-based diet
Source: Anim Nutr. 2023 Mar 7;13:302–12. doi: 10.1016/j.aninu.2023.02.011 (PMC10165182; doi:10.1016/j.aninu.2023.02.011)
Supplement: Multimedia component 1 [file mmc1.docx]

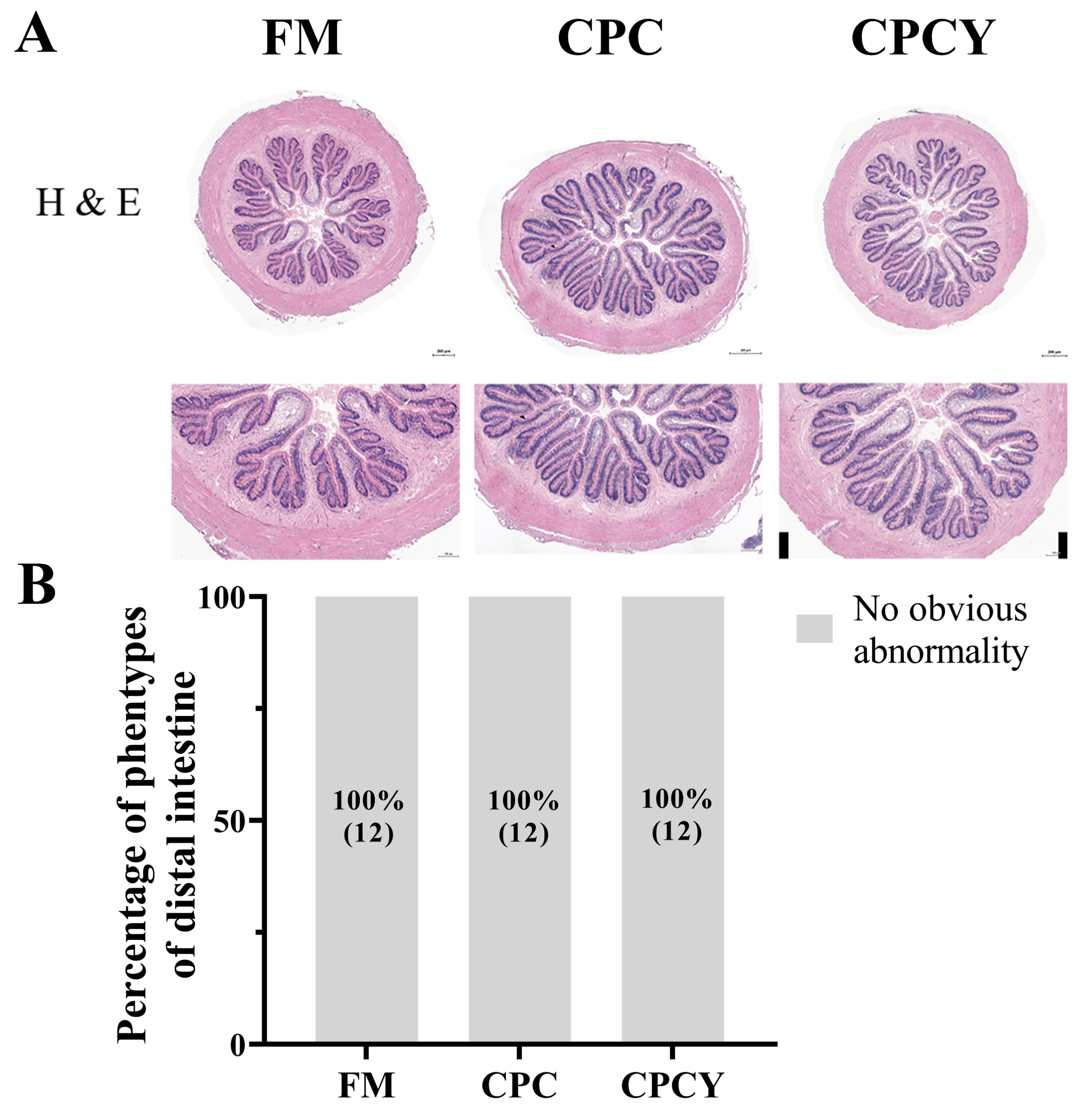


**Fig. S1.** Light microscopy of distal intestine morphology of largemouth bass fed experimental diets. (A) hematoxylin and eosin (H & E). (B) Histopathological examination of distal intestine between groups (*n* = 12). FM = fish meal diet; CPC = cottonseed protein concentrate diet; CPCY = CPC diet + 800 mg/kg multi-strain yeast fractions (MsYF).


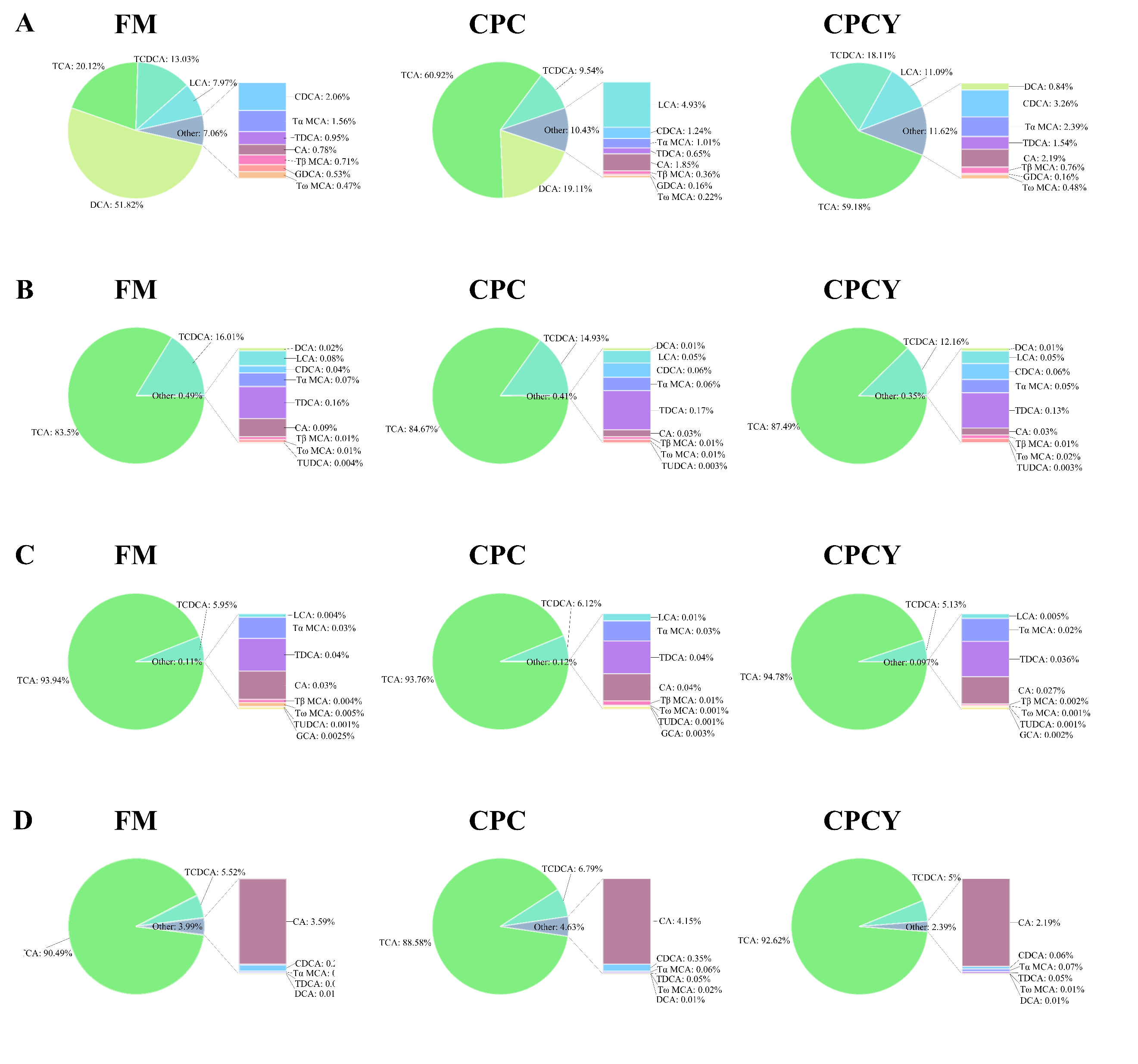


**Fig. S2.** Bile acids composition in (A) plasma, (B) liver, (C) gallbladder, and (D) distal intestinal chyme of largemouth bass at 10-week feeding trial. FM = fish meal diet; CPC = cottonseed protein concentrate diet; CPCY = CPC diet + 800 mg/kg MsYF; T*α* MCA = tauro-*α*-muricholic acid; T*β* MCA = tauro-*β*-muricholic acid; T*ω* MCA = tauro-*ω*-muricholic acid; TCA = tauro-cholic acid; TCDCA = tauro-chenodeoxycholic acid; TUDCA = tauro-ursodeoxycholic acid); TDCA = tauro-deoxycholic acid; GCA = glycol-cholic acid; GDCA = glycol-deoxycholic acid; CA = cholic acid; CDCA = chenodeoxycholic acid; DCA = deoxycholic acid; LCA = lithocholic acid.
