# Supplementary material for: Effects of paraprobiotics on bile acid metabolism and liver health in largemouth bass (Micropterus salmoides) fed a cottonseed protein concentrate-based diet
Source: Anim Nutr. 2023 Mar 7;13:302–12. doi: 10.1016/j.aninu.2023.02.011 (PMC10165182; doi:10.1016/j.aninu.2023.02.011)
Supplement: Multimedia component 2 [file mmc2.docx]

**Table S1.** Amino acid composition of experimental diets.

| **Item** | **Diet**^1^ |  |  |  |
| --- | --- | --- | --- | --- |
|  | **FM** | **SPC** | **CPC** | **CPCY** |
| Amino acids, g/kg as-is crude protein | | | | |
| Essential amino acid, EAA | | | | |
| Lysine | 70.2 | 70.4 | 72.0 | 72.1 |
| Threonine | 39.5 | 40.0 | 40.9 | 41.5 |
| Valine | 50.6 | 49.7 | 48.2 | 46.7 |
| Methionine | 23.3 | 23.1 | 21.9 | 21.9 |
| Isoleucine | 37.1 | 39.0 | 30.8 | 30.1 |
| Leucine | 78.3 | 81.1 | 67.5 | 67.1 |
| Arginine | 54.2 | 64.1 | 91.8 | 90.5 |
| Phenylalanine | 44.9 | 50.5 | 53.9 | 52.4 |
| Histidine | 33.7 | 31.9 | 31.8 | 31.5 |
| ƩEAA^2^ | 431.7 | 449.8 | 458.8 | 453.9 |
| Nonessential amino acid (NEAA) |  |  |  |  |
| Proline | 44.7 | 50.3 | 44.8 | 42.3 |
| Glycine | 54.2 | 44.7 | 42.5 | 42.4 |
| Cystine | 10.6 | 13.6 | 13.8 | 13.4 |
| Alanine | 58.4 | 48.7 | 44.2 | 44.7 |
| Serine | 40.4 | 48.8 | 40.4 | 41.8 |
| Aspartic acid | 90.5 | 105.0 | 89.2 | 89.6 |
| Glutamic acid | 145.3 | 173.0 | 178.8 | 178.4 |
| ƩNEAA^3^ | 444.2 | 484.1 | 453.7 | 452.6 |
| ƩAA^4^ | 875.9 | 933.9 | 912.4 | 906.4 |
| ƩEAA:ƩAA | 0.49 | 0.48 | 0.50 | 0.50 |

^1^ FM = fish meal diet; SPC = soy protein concentrate diet; CPC = cottonseed protein concentrate diet; CPCY = CPC diet + 800 mg/kg multi-strain yeast fractions (MsYF);

^2^ ΣEAA: sum of essential amino acids;

^3^ ΣNEAA: sum of non-essential amino acids;

^4^ ƩAA: sum of total amino acids.

**Table S2. Primer pair sequences for real-time PCR.**

| **Gene** | **Primer** | **Sequence 5′-3′** | **Amplicon size , bp** | **E-values, %** | **Tm, °C** | **Sources** |
| --- | --- | --- | --- | --- | --- | --- |
| *ef-1α* | F | TGCTGCTGGTGTTGGTGAGTT | 147 | 102.8 | 60.4 |  |
|  | R | TTCTGGCTGTAAGGGGGCTC |  |  |  |  |
| *pparγ* | F | CCTGTGAGGGCTGTAAGGGTTT | 103 | 100.2 | 59.0 |  |
|  | R | TTGTTGCGGGACTTCTTGTGA |  |  |  |  |
| *fasn* | F | TGTGGTGCTGAACTCTCTGG | 121 | 102.1 | 57.5 |  |
|  | R | CATGCCTAGTGGGGAGTTGT |  |  |  |  |
| *acc-1* | F | ATCCCTCTTTGCCACTGTTG | 121 | 102.2 | 57.5 |  |
|  | R | GAGGTGATGTTGCTCGCATA |  |  |  |  |
| *dgat1* | F | CACGCCTCTTCTTGGAGAAC | 176 | 105.3 | 58.5 |  |
|  | R | AATGGTACCCACAGCCAGAC |  |  |  |  |
| *lpin1* | F | TCCTACGTTCCCGAGAGAAA | 136 | 98.8 | 58.5 |  |
|  | R | TACGAGGGAACCACTTCCTG |  |  |  |  |
| *hmgcr* | F | GAGGCCACCATACCTGCTAA | 123 | 98.9 | 60.0 |  |
|  | R | AAATCCACCGATGCTACCTG |  |  |  |  |
| *pparα* | F | CCACCGCAATGGTCGATATG | 144 | 104.3 | 59.0 |  |
|  | R | TGCTGTTGATGGACTGGGAAA |  |  |  |  |
| *cpt1α* | F | CATGGAAAGCCAGCCTTTAG | 128 | 98.8 | 60.0 |  |
|  | R | GAGCACCAGACACGCTAACA |  |  |  |  |
| *atgl* | F | CCATGATGCTCCCCTACACT | 176 | 99.1 | 58.0 |  |
|  | R | GGCAGATACACTTCGGGAAA |  |  |  |  |
| *hsl* | F | ATCAGAGCTGGAGCACCCTA | 122 | 99.3 | 60.0 |  |
|  | R | GCAGAGGAGAGCAGAAAGGA |  |  |  |  |
| *mgl* | F | AAGGTTTTTCTGGCGAAGGT | 130 | 96.8 | 58.0 |  |
|  | R | CGTGGAAGTTCAGCTCATCA |  |  |  |  |
| *cyp7a1* | F | CTGGGCTTCACAGGCTAACACC | 153 | 102.2 | 60.4 |  |
|  | R | TTCAGTGTGGGGTCGTTGGG |  |  |  |  |
| *cyp8b1* | F | TAGACAGCGGCAACCAGGAG | 120 | 100.8 | 60.4 |  |
|  | R | CCGTGCTTTTGTTTCATCCTATC |  |  |  |  |
| *cyp27a1* | F | ATGCCCGTGTCACTGTTG | 176 |  |  | Xie et,al(2020) |
|  | R | GTGCGGCTTGGACTTCTC |  |  |  |  |
| *cyp7b1* | F | CGACAAAGCAGCTCATCACTT | 220 | 92.8 | 57.0 |  |
|  | R | CTCCAGATAAAGAAGCTTGTCCA |  |  |  |  |
| *fxr* | F | TAAAAGGCTGCGAAAGAACACC | 103 | 100 | 60.4 |  |
|  | R | GGTGGTTGATGTGACCTGTTTGT |  |  |  |  |
| *shp* | F | AACCAACTCTTGCTGAAGTCCAC | 149 | 103.1 | 60.4 |  |
|  | R | TTCAACAAACGACAAGGCACTC |  |  |  |  |
| *besp* | F | GTCATCTCCGCCGCCAAGAA | 211 | 91.0 | 60.5 |  |
|  | R | GCACAGTCTTCTCGCTCTCAGT |  |  |  |  |
| *ostβ* | F | CGACCACATGGAAGTTTGTG | 169 | 93.0 | 60.5 |  |
|  | R | ACGACCAACATCTCCTCACC |  |  |  |  |
| *i-babp* | F | CAAAGAGTCCAGCACCTTCC | 178 | 99.1 | 60.5 |  |
|  | R | CAGAGCTCTTGTGGTGGTCA |  |  |  |  |
| *slc10a2* | F | CTGGACTTCCAACAGCACAA | 161 | 90.4 | 60.5 |  |
|  | R | TGAGAGCAAATCCTGCAATG |  |  |  |  |
| *slc10a4* | F | CGAGGCTGGTGTTTTAATCC | 173 | 94.0 | 60.5 |  |
|  | R | ACATGATGGTGTTTGGGTCA |  |  |  |  |
| *slc10a7* | F | CCGTCTCGCTCATCTTCTTC | 204 | 91.1 | 60.5 |  |
|  | R | TGCAGCTCACCGTCTGTAAC |  |  |  |  |
| *mdr1* | F | ACGTGTGCTTTGACAACGTC | 178 | 94.1 | 60.5 |  |
|  | R | CTCCCCTCTCTGGCATCATA |  |  |  |  |

*ef-1α* = elongation factor 1α; *ppar* = peroxisome proliferators-activated receptors; *fasn* = fatty acid synthase; *acc-1* = acetyl CoA carboxylase-1; *dgat1* = diacylglycerol acyltransferase 1; *lpin1* = lipid phosphate phosphohydrolase 1; *hmgcr* = 3-hydroxy-3-methyl glutaryl coenzyme A reductase; *cpt1α* = carnitine palmitoyltransferase 1α; *atgl* = adipose triglyceride lipase; *hsl* = hormone-sensitive lipase; *mgl* = monoacylglycerol lipase; *cyp7a1* = cytochrome P450 7A1; *cyp8b1* = cytochrome P450 8B1; *cyp27a1* = cytochrome P450 27A1; *cyp7b1* = cytochrome P450 7B1; *fxr* = farnesoid X receptor; *shp* = nuclear receptor subfamily 0 group B member 2; *besp* = bile salt export pump; *ostβ* = organic solute transporter β; *i-babp* = ileal bile acid binding protein; *slc10a* = sodium/bile acid cotransporter; *mdr1* = multidrug resistance protein 1; F = forward primer; R = reverse primer.
